# Untapped Potential of Side Stream Products from the Industrial Processing of Fruits: The Biosorption of Anthocyanins on Raspberry Seeds

**DOI:** 10.3390/foods13152334

**Published:** 2024-07-25

**Authors:** Dragana Dabić Zagorac, Milica Sredojević, Milica Fotirić Akšić, Ivanka Ćirić, Biljana Rabrenović, Ilinka M. Pećinar, Maja Natić

**Affiliations:** 1 Innovative Centre of the Faculty of Chemistry, University of Belgrade, Studentski Trg 12-16, 11158 Belgrade, Serbia; ddabic@chem.bg.ac.rs (D.D.Z.); pantelicm@chem.bg.ac.rs (M.S.); 2 Faculty of Agriculture, University of Belgrade, Nemanjina 6, 11080 Belgrade, Serbia; fotiric@agrif.bg.ac.rs (M.F.A.); biljanar@agrif.bg.ac.rs (B.R.); ilinka@agrif.bg.ac.rs (I.M.P.); 3 Faculty of Chemistry, University of Belgrade, Studentski Trg 12-16, 11158 Belgrade, Serbia

**Keywords:** biosorbent, defatted raspberry seed, revalorization, anthocyanins, LC-MS, FTIR, Raman

## Abstract

Natural biosorbents from agricultural side stream products are being investigated due to their large surface area and capacity for various compounds. The aim of the present work was to investigate the raspberry seeds and their sorption potential in the recovery of natural pigments. The experiment included raspberry seed and a liquid by-phase rich in anthocyanins initially collected during the depulping of the raspberry seed material. Biosorption was monitored by LC-MS analysis of the anthocyanins and by the total anthocyanin content (TAC) before and after biosorption. Cyanidins predominated in the seed material, followed by pelargonidins and peonidins. The efficiency of biosorption was examined by comparing the percent of removal. The heterogeneous polymer structure of the biosorbent, which consists mainly of lignin, cellulose, and hemicellulose, was characterized by Fourier Transform Infrared Spectroscopy (FTIR) and Raman Spectroscopy (RS). The FTIR spectra of raw and defatted seed indicated functional groups involved in biosorption and principal component analysis (PCA) performed on Raman spectra pointed to differences among biosorbents. The developed strategy for the valorization of raspberry seeds in the recovery of natural colorants was shown to be effective, with recoveries from 49 to 88 percent of total anthocyanins.

## 1. Introduction

The design of an analytical approach in chemistry should go through several important phases in solving multidisciplinary tasks in order to ensure the basic principles of green solutions, ecology, and environmental sustainability, which are the main keywords of the circular economy today. Production and consumption models based on the reuse of existing materials are one of the ways to extend the life cycle of products. In the agricultural sector, which operates according to a linear model, large quantities of waste are produced. In addition to the waste and loss of food, the side streams of food have a significant impact on the environment. For responsible consumption and production, it is therefore important to avoid food waste and loss and to use green biotechnologies to utilize natural renewable sources.

Raspberries, as a fruit species, are very popular among producers because they require less in planting and maintaining orchards and bring quick financial returns, resulting in high profits [1]. Raspberries are very popular with consumers because of their enjoyable and refreshing color, delicious aroma and taste, and low-calorie content. They are also exceptional sources of health-promoting compounds such as sugars, organic acids, phenolics, anthocyanins, carotenoids, tannins, vitamins, dietary fibers, and minerals [2,3,4,5]. This type of food is effective in eliminating free radicals and reactive oxygen species that in most cases are the cause of chronic diseases [6]. They are consumed as table fruit but are very often sold as frozen, canned, or dried and are used to make raspberry puree and juice concentrate. According to FAOStat [7], global raspberry production in 2022 amounted for ~948,000 tons, of which ~617,000 tons were produced in Europe. The largest raspberry producers in Europe in 2022 were Russia (212,300 tons), Serbia (116,093 tons), and Poland (104,900 tons). Considering that the seeds account for up to 5% of the fresh weight of the fruit and taking into account global production, the processing of raspberries could result in tons of bio-waste. Furthermore, after oil extraction from the seeds, just over 90% is still available for recycling.

In addition to the seed, pomace is also a side-stream product of the industrial processing of berries. Raspberry pomace is a rich source of anthocyanins. As natural colorants, anthocyanins are considered safe and healthy compared to the artificial food colorants commonly used. Given the amount of fruit and vegetable secondary products derived from the production process, the relevance of novel and innovative extraction technologies for pigments from the agri-food industry is not surprising. Conventional methods of extracting natural pigments from food waste are time-consuming, expensive, and unsustainable.

Natural biosorbents have a large surface area and a good capacity for various compounds. The most studied materials come from agricultural bio-waste. Raspberry seeds from the fruit industry can be categorized as a low-cost and highly available biosorbent; the previous studies were related to the removal or recovery of heavy and toxic metals from wastewater [8,9]. The recovery of important phytochemicals can also be found in the literature and several studies have focused on the biosorption of anthocyanins [10,11]. However, most studies refer to anthocyanins from grape pomace [12], which is not surprising considering the widespread use of this industry.

Therefore, we designed the experiment to investigate two side stream products that are commonly obtained during raspberry processing: the seeds and the liquid by-phase, which is rich in anthocyanins. The main goal was to investigate the sorption potential of the seed material for the recovery of the natural pigments. The potential of biosorption was evaluated for both raw seed and defatted raspberry seed material. The recovery of anthocyanins from liquid by-phase fractions was evaluated by LC-MS analysis. Fourier transform infrared (FTIR) and Raman spectroscopy were used to analyze the seed material and the biosorption process. As far as we are aware and the literature search has revealed, none of the studies have investigated raspberry seeds and liquid by-phase as side stream products resulting from the same industrial processing. The approach is cost-effective, time-saving, and technically feasible and could easily be implemented as a solution for the recovery of and reduction in side stream products.

## 2. Materials and Methods

All chemicals used were of analytical grade. Methanol, acetonitrile (both HPLC grade), formic acid, and potassium chloride were purchased from Merck (Darmstadt, Germany). Acetic acid and ethanol were purchased from Zorka-Pharma (Šabac, Serbia). Hydrochloric acid (37%) was purchased from Fisher Scientific (Loughborough, UK). Acetate buffer was prepared from sodium acetate (99%) and glacial acetic acid (98%), both from Sigma-Aldrich (Steinheim, Germany). Syringe filters (13 mm, PTFE membrane 0.45 μm) were purchased from Supelco (Bellefonte, PA, USA). The Thermo Fisher TKA MicroPure (Waltham, MA, USA) water purification system was used to obtain ultrapure water (0.055 µS/cm) for the preparation of aqueous solutions of the blanks and standards. Anthocyanin standards were purchased from Extrasynthese (Genay, France).

### 2.1. Sample Preparation

**Figure 1:**
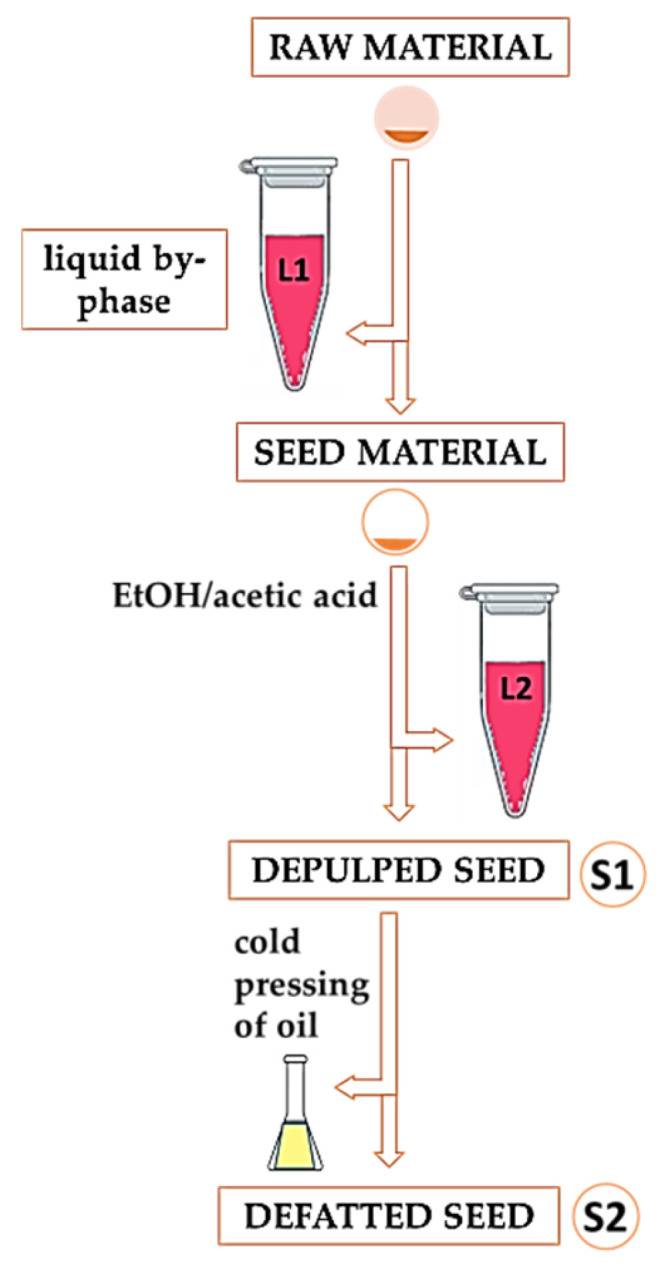
Preparation of seed material for the biosorption experiment.

Raspberry seeds were collected from a small juice manufacturer in Serbia. The integrated production was organized on Kopaonik Mountain (central part of Serbia), at 700 m of altitude. The standard cultural practice was applied in the orchard. The fully ripened fruits of the Willamette raspberry cultivar were used for juice production. The experiment was designed to use both depulped seed (S1) and defatted seed cake (S2), to recover anthocyanins from the pulp residue. For this purpose, the raspberry seed sample was processed in such a way that both the solid phase and the liquid by-phase phase were fully utilized in each step of the entire procedure. In other words, no filtrates or solid residues were discarded. The workflow for the processing of samples S1 and S2 as well as the liquid by-phases L1 and L2 is shown in Figure 1.

The first experimental phase comprised the steps necessary to collect the anthocyanin-rich pulp residue. In total, 5 mL of the first liquid by-phase (L1) was collected during the thawing of 100 g of seeds, while the second (L2) was obtained when the remaining seeds were washed with 500 mL acidified ethanol. This organic solvent mixture was chosen based on safety, economic, and ecological parameters. Such a procedure contributes to greenness and is expected to have a higher EcoScale score. In addition, the application of these solvents is facilitated, bearing in mind that both are freely available, even to small juice producers. The liquids obtained were stored at a temperature of 4 °C until further biosorption experiments. The depulped seed was air-dried for about 48 h and then dried in an oven at 100 ± 5 °C for 24 h. Dried depulped seed material (S1) was used for further experiments to obtain defatted seeds (S2). The seeds were pulverized and sieved through a 100-μm sieve and stored in an airtight container to obtain the oil fraction and defatted seed material. The cold pressing of the raspberry seeds was described in detail in a previously published article [13]. For further experiments, the defatted seeds were ground on a Grindomix GM200 (Retsch, Haan, Germany), sieved on a sieve with a pore size of 250 μm (Retsch, Haan, Germany), and stored in closed polyethylene bags at room temperature until analysis. 

### 2.2. Biosorption Experiment

In this part of the study, an attempt was made to use seeds as biosorbent and to investigate their efficiency. Two seed samples were used, depulped seed (S1) and defatted seed (S2), and both were characterized before and after biosorption using infrared techniques. A sorption study was performed using 0.5 g of biosorbent (S1 and S2) and 5 mL of the anthocyanin-rich extract collected during the preparation of the seed material for oil extraction, i.e., the liquid by-phase (L1 and L2, Figure 1). The mixtures of biosorbent and solution rich in anthocyanins were left for 24 h in the dark at room temperature. The sorption of L1 and L2 extracts on depulped seed S1 yielded two filtrates, L1S1 and L2S1. Similarly, after sorption on defatted seed S2, two filtrates were collected (L1S2 and L2S2). 

The efficiency of adsorption (%Removal, Equation (1)) was evaluated by the relative decrease in total anthocyanin content (TAC) after sorption and by the quantitative data of characteristic anthocyanins determined by LC-MS. The adsorption efficiency was calculated for each anthocyanin (mg/L) and for the total anthocyanin content (mg cya-3-glu/L), where C_o_ and C_e_ are the initial and final concentration.

(1) $$\%Removal=\frac{C_{0}-C_{e}}{C_{0}}\times 100\%$$

### 2.3. Determination of the Total Anthocyanin Content

The total anthocyanin content (TAC) was determined using the pH-differential method, which allows accurate and rapid measurement of the total anthocyanins. As described in [14], the extracts obtained were diluted with buffers of pH 1.0 (KCl, 0.025 mol/L) and pH 4.5 (NaOAc/HOAc, 0.4 mol/L). After equilibration for 15 min, the absorbances were measured at 510 and 700 nm and the absorbance value of the diluted sample (A_tot_) was calculated as follows:

(2) $$A_{\mathrm{tot}}={\left(A_{510}-A_{700}\right)}_{{\mathrm{pH}}_{1.0}}-{\left(A_{510}-A_{700}\right)}_{\mathrm{pH}4.5}$$

The concentration of monomeric anthocyanin pigments, expressed as TAC, was calculated using the following equation:

(3) $$\mathrm{TAC}=\frac{A_{\mathrm{tot}}\times MW\times DF\times 1000}{\left(\varepsilon \times l\right)}$$

where MW was the molecular weight of cyanidin-3-O-glucoside (MW = 449.2 g/mol), DF-the dilution factor, l-the cuvette path length (1 cm), ε-molar absorptivity of cyanidin-3-O-glucoside (ε = 26,900 L/(mol cm)). The results of three replicates were expressed in mg of cyanidin-3-O-glucoside equivalents per L of extracts (mg cya-3-glu/L). 

### 2.4. Determination of the Anthocyanin Profile Using UHPLC-DAD MS/MS

Anthocyanins were quantified using a Vanquish UHPLC system equipped with a diode array detector (DAD) coupled to TSQ Fortis triple-quadrupole mass spectrometer (ThermoFisher Scientific, Bremen, Germany), with a heated electrospray ionization (HESI) source operated in the positive ionization mode. The column used for the analytical separation was an Accucore™ aQ C18 Polar endcapped HPLC Column (100 mm × 2.1 mm) with 2.6 μm particle size (ThermoFisher Scientific, Bremen, Germany). The column was kept at 40 °C throughout the analysis. Xcalibur software (version 2.2) was used for instrument control and TraceFinder software (version 5.2) for data acquisition and processing. Aqueous formic acid solution at 0.1% (A) and acetonitrile 0.1% formic acid (B) were the components of the mobile phase used for gradient elution. The flow rate was 0.25 mL/min with the eluent gradient listed in Table 1. 

Anthocyanins were identified by direct comparison with commercial standards and the concentrations of each compound were estimated by calculating the peak areas and are expressed in mg/L. All experiments were performed in triplicate. The regression parameters obtained for anthocyanin standards using LC-MS analysis are presented in Table S1. The analyses were performed in the accredited laboratory AEROLAB d.o.o. (Belgrade, Serbia), which specializes in environmental testing and consulting services.

### 2.5. FTIR Spectroscopy

The FTIR spectra of the depulped seed (S1) and the defatted seed (S2) before and after biosorption were recorded using an ATR-FTIR spectrometer (Nicolet iS5 FTIR with iD7 Diamond ATR, Thermo Scientific). The measurements were performed in the spectral range 4000–600 cm^−1^ with a resolution of 4 cm^−1^. In total, 16 average scans were recorded. The processing of the spectra was conducted by the OMNIC software Version 9.

### 2.6. Raman Spectroscopy

Raman microspectroscopy focused on the measurement of depulped seed (S1) and defatted seed (S2), before and after biosorption. The spectra were recorded using the XploRA Raman spectrometer (Horiba Jobin Yvon, Montpellier, France). In seeds, Raman scattering was excited by a laser with a wavelength of 785 nm equipped with 600 lines mm^−1^ grating; spectra were recorded with an exposure time of 5 s and accumulated from five scans. The spectral resolution was about 3 cm^−1^ and autocalibration was performed each time before recording the spectra through the 520.47 cm^−1^ line of silicon. To assess the possible inhomogeneity of the sample, a maximum of 10 Raman spectra were recorded from each sample. The identification of the main bands was based on the literature data. The spectra were pre-processed using Spectragryph software Version 1.2.8 [15]. The spectra were baseline-corrected using Savitzky–Golay filters with seven points and a second-order polynomial function was used for spectra smoothing. PCA was performed using the PAST software Version 3.26b [16]. 

## 3. Results

### 3.1. LC-MS Analysis of Liquid By-Phase Anthocyanins

Structurally, anthocyanins belong to the flavonoids and occur in the form of anthocyanidin glycosides and acylated anthocyanins, polar water-soluble compounds. Of all of the anthocyanins found in nature, only cyanidin, delphinidin, petunidin, peonidin, pelargonidin, and malvidin are widely distributed. In berries, cyanidins predominate, followed by pelargonidins and peonidins. The efficiency of anthocyanin extraction depends on many factors, such as temperature, luminosity, composition of solvents, pH, and extraction time. These external conditions should be controlled to prevent their degradation [17]. 

Table 2 shows the data of LC-MS quantification of anthocyanins in liquid by-phases before and after the sorption experiment together with the results of the total anthocyanin content (TAC). The efficiency is expressed in brackets by the percent of recovery (%Recovery). Quantification was based on 12 standards of anthocyanin glycoside derivatives. The LC-MS results showed the presence of almost all anthocyanins analyzed in the liquid by-phases L1 and L2, with the exception of cyanidin-3,5-di-O-glucoside, delphinidin-3-O-glucoside, and malvidin-3,5-di-O-glucoside, which were not found. 

The most abundant in L1 was cyanidin-3-O-sophoriside (28.4 mg/L), followed by cyanidin-3-O-glucoside (19.6 mg/L) and pelargonidin-3-O-rutinoside (16.0 mg/L). In the case of L2, which was collected in the next step when the remaining seeds were washed with acidified ethanol, the concentration profile was slightly different and cyanidin-3-O-glucoside was the most abundant with a concentration of 15.1 mg/L. The differences in the abundance of anthocyanins that occur between the two liquid by-phases could be associated with the polarity and acidity of the solvents used for the collection of the L2 phase. As expected, the concentrations of individual anthocyanins were lower in this phase compared to phase L1, which was also found for the TAC values. Other less abundant anthocyanins showed a similar trend in terms of the order of concentrations. 

Looking at the decrease in concentration of individual anthocyanins in both sorption experiments, several important conclusions can be drawn based on Table 2. Cyanidin-3-O-arabinoside, which was found in the lowest concentration, was completely removed. Pelargonidin-3-O-rutinoside was completely removed from L2, whereas this was not the case in the sorption experiment of L1, as its concentration decreased fourfold.

From the data presented in Table 2, the ability of biosorbent to extract anthocyanins was calculated using Equation (1). The sorption efficiency based on the individual anthocyanins and total anthocyanins (TAC) provides a general insight into the entire experiment. Based on the calculated values, it is clear that significant removal of TAC occurred when defatted seed served as a biosorbent (%Removal, 88). In addition, the %Removal values in the sorption experiment of total anthocyanins in the L1 phase were also significant, as the sorption on depulped seed (S1) was 70%, while the value on defatted seed (S2) was slightly lower (66%). The lowest value was found for sorption TAC from the liquid by-phase L2 (acidified ethanol). It is evident from Table 2 that the used biosorbents showed different degrees of sorption of individual anthocyanins. Both biosorbents, depulped and defatted seeds, showed the lowest efficiency for the sorption of malvidin-3-O-glucoside and peonidin-3-O-glucoside. Depulped seed proved to be a very efficient biosorbent for cyanidin-3-arabinosides (100%), cyanidin-3-O-glucosides (96% and 98%), and pelargonidin-3-O-glucosides (90% and 91%). Cyanidin-3-O-arabinoside and pelargonidin-3-O-rutinoside, from both liquid phases L1 and L2, were completely adsorbed on defatted seeds (%Removal, 100). 

A review of the literature indicates that the issue is important and current. It is evidenced by numerous works aimed at reducing the waste generated in the food production industry. As an example, we single out the works published recently by Barroso et al. [18,19]. A similar idea stood behind their extensive investigation of jabuticaba byproducts and industrial waste. The anthocyanin-rich extract used for the adsorption studies was the jabuticaba peel extract while the jabuticaba peel residue served as the biosorbent. Both studies revealed the high adsorption capacity of biosorbents, based on the measurements of the concentration of cyanidin-3-glucoside [18] and anthocyanins extracted by one-step extraction and the purification method [19].

### 3.2. Characterisation of Biosorbent

#### 3.2.1. FTIR Analysis

**Figure 2:**
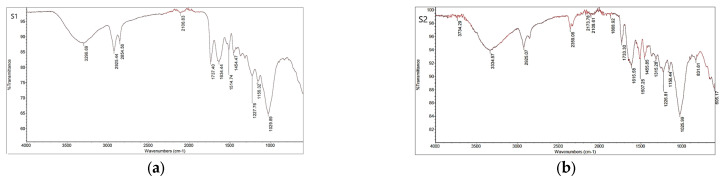
FTIR spectra of the seed material before biosorption experiments: (**a**) S1-depulped seed; (**b**) S2-defatted seed.

Seed material is generally valued as a sorption phase because it contains functional groups of organic compounds that can interact with each other via absorption mechanisms. To determine the functional groups responsible for the biosorption of anthocyanins, an FTIR analysis was performed. Figure 2 shows the spectra of the tested seed samples before the biosorption experiment as well as the depulped seed (Figure 2a) and the defatted seeds (Figure 2b) before the absorption of anthocyanins. The FTIR spectra of the seeds showed a series of absorption peaks, indicating the complex nature. In general, the spectra show a similar absorption pattern, while slight differences were observed in the fingerprint regions of the spectra, indicating the specific chemical structure of the biosorbents.

**Figure 3:**
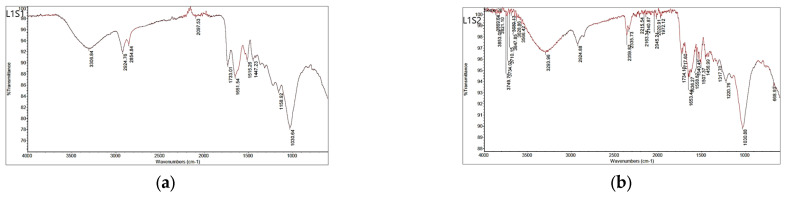
FTIR spectra of the seeds after biosorption of anthocyanins: (**a**) L1S1-raw seed and (**b**) L1S2-defatted seed.

**Figure 4:**
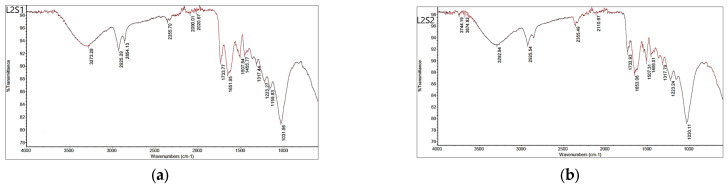
FTIR spectra of the seeds after biosorption of anthocyanins: (**a**) L2S1-depulped seed and (**b**) L2S2-defatted seed.

The main bands refer to the complex polymeric structure of the seed, which consists mainly of cellulose, hemicellulose, and lignin, as well as other less abundant compounds in the seed (e.g., proteins, lipids, and aromatic compounds). The most important seed compound is lignin [20]. Broad bands around 3300 cm^−1^ were present in all FTIR spectra, originating from stretching vibrations of the O-H groups (lignin). The absorption bands at 2925 cm^−1^ and 2854 cm^−1^ in the spectra of the seeds originate from the C-H stretching vibrations of the aliphatic groups CH_2_ and CH and did not change after sorption. The band at 1737 cm^−1^ in the spectrum of depulped seed S1 (Figure 2a), which is assigned to the C=O stretching vibrations of the carbonyl group, was slightly lower (1733 cm^−1^) in the spectrum after sorption (Figure 3a and Figure 4a). The band corresponding to the O-H bending vibrations (polysaccharide) was at 1634 cm^−1^ in the spectra of the seed S1, while in the spectra after sorption, the bands were at 1651–1653 cm^−1^ (Figure 3 and Figure 4). In all spectra, bands around 1030 cm^−1^ are due to the C-O, C=C, and C-C-O stretching vibrations of cellulose, hemicellulose, and lignin, except in the spectra of the defatted seed, where the corresponding band was slightly lower (1026 cm^−1^). The bands around 1454 cm^−1^ in all spectra originate from CH_2_ scissoring vibrations, most likely from cellulose. Fourier transform infrared spectra show that some shifts occur, most likely associated with changes in the chemical environment of the functional groups caused by molecular interactions during sorption.

#### 3.2.2. Raman Spectroscopy

**Figure 5:**
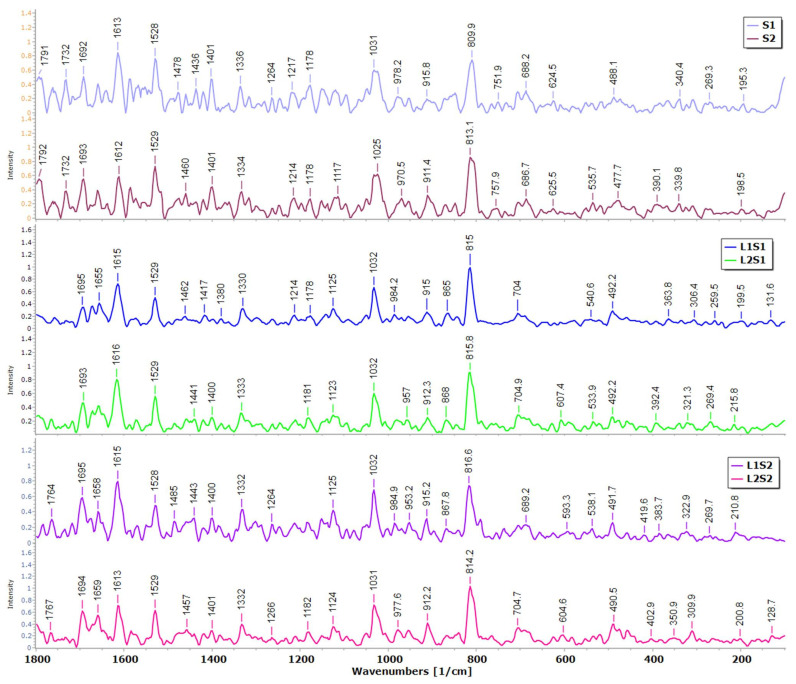
The preprocessed Raman spectra of seed samples in the range of 100 cm^−1^ to 1800 cm^−1^.

We also investigated whether Raman spectroscopy can be used to unambiguously identify the chemical composition of seeds and possible changes through experimentation. We collected 60 spectra of 6 sample types (S1, S2, L1S1, L2S1, L1S2, and L2S2). The Raman spectra of the seed samples (Figure 5) can be considered as fingerprints of the samples and are closely related to their chemical composition. According to the Raman spectra, the raspberry seeds exhibit vibrational bands that can be assigned to phenolic acids (815 cm^−1^), polyphenols (1032, 1400, 1528, 1615, and 1655 cm^−1^), proteins or fatty acids (1264, 1443, and 1650 cm^−1^), and carbohydrates (915, 984, and 1125 cm^−1^). Band position and intensity (Figure 5 and Table 3) of the seed samples indicate the predominance of phenolic acids, anthocyanidins, and other polyphenols [21,22,23]. Differences in the position and intensity of the band. 

Differences in the position and intensity of the band at around 815 cm^−1^ indicate that the biosorption processes of a liquid by-phase L2 could alter the phenolic acid content in the seed after sorption, i.e., compared to seed sample S1, the intensity of this band is higher in L2S1 than in S1. The spectra with the higher intensity of the band at around 1528 and 1613 cm^−1^ could indicate a slightly higher content of polyphenols in L1S2 than S2. Compared to S1 and S2, samples L1S1 and L1S2 show a higher intensity of the band at approximately 1125 cm^−1^, which could indicate a higher concentration of carbohydrates. The band at 1732 cm^−1^ could indicate fatty acid esters, which are only found in the seed samples S1 and S2 [24].

In particular, biological samples, which are very heterogeneous, often show several overlapping bands associated with a large number of metabolites (such as anthocyanidins and polyphenols), making it difficult to assign a specific band to a specific compound. However, the differences between the seed samples in terms of their chemical composition cannot be determined from the general characteristics of the Raman spectra alone. In order to correctly classify the differences between the seeds before and after the sorption experiments and to allow objective discrimination between the samples, a multivariate analysis based on PCA was performed.

**Figure 6:**
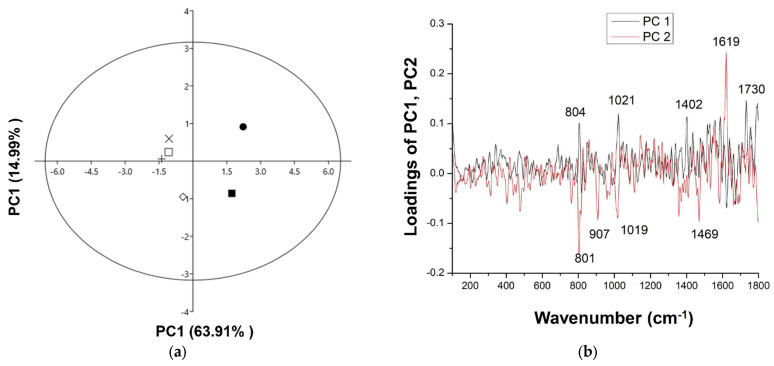
PCA applied to the data obtained from Raman spectra of raspberry seed samples: (**a**) score plot and (**b**) loading plots. (S1-closed cycle, L1S1-plus, L2S1-open square, S2-closed square, L1S2-exs, L2S2-rhombus).

Prior to the multivariate analysis, all data were preprocessed. The first PCA model created for all seed samples resulted in three principal components that explained 89.48% of the total data variance. The first principal component (PC1) explained 63.91% of the total data variance, while the second (PC2) accounted for 14.99%. The mutual projections of the factor scores and their loadings for the first two PCs are shown in Figure 6.

The score plot (Figure 6a) shows the existence of two groups of items along the PC1 axis. S1 and S2 belong to the first group, while all of the others form the second group. The corresponding loading plot represents the relationship between the variables and can be used to identify the variables with the highest contribution to object positioning in the score plot. The loading plot (Figure 6) shows that the variables with the highest positive contribution along the PC1 axis corresponded to the signals at 804, 1021, 1402, and 1730 cm^−1^. These variables are due to the polysaccharide (band at 804 cm^−1^), anthocyanidins (bands at 1021 and 1402 cm^−1^), and fatty acids ester content [22,32] and are responsible for the differences between the seed samples before biosorption (S1 and S2) and other samples that adsorbed anthocyanins from L1 and L2. 

The variables that potentially had the highest positive influence on the separation along PC2 (Figure 6b) corresponded to the signals at 1619, 801, 907, 1019, and 1469 cm^−1^. The differences between the S1, L1S1, and L2S1, compared to S2 and L2S2, depend mainly on v (C-O-O) and v (C=C) aromatic vibration of phenylpropanoids [28,33]. A high-intensity positive signal at 1619 cm^−1^ and lower and negative intensity loadings at 801, 907, and 1019 cm^−1^ are related to the presence of carbohydrates, phenylpropanoids [28], and probably a lower concentration of lipids in S2 and L2S2 than in other samples (indicated loading at 1468 cm^−1^) [34].

## 4. Conclusions

Our aim was to propose a simple easy-to-use process that can be applied to any plant material with high contents of anthocyanins or other natural compounds with interesting physicochemical and health-related properties for the food, cosmetics, and pharmaceutical industries. Our research focuses on sustainability, as the processing of fruit in the juice industry leaves tons of seeds and pomace behind. This approach is a valuable economical solution that can be implemented without high-tech solutions, which is particularly useful for small fruit juice producers who also have a waste problem and where large amounts of peel and pomace are often left behind. 

The results presented herein show that both the seeds and the residue from cold pressing of the defatted seed cake are effective. Such biosorbents are promising alternatives and their utilization could be considered as a sustainable approach to reduce agricultural side stream products. Furthermore, we believe that raspberry seed could serve as biosorbent with various applications such as extraction, filtration, and purification of liquid media.

We consider this research to be an example of sustainable agriculture that demonstrates how renewable biological resources can be used to produce other valuable products. All activities are at a high ecological level and support the bioeconomy through new value chains.

## Figures and Tables

**Table 1:** HPLC gradient conditions.

Time (min)	Mobile Phase A (%)
0.0–1.0	95
1.0–17.0	95–60
17.0–17.1	60–5
17.1–18.0	5
18.0–18.1	5–95

**Table 2:** LC-MS quantification of anthocyanins in the liquid by-phase before and after sorption experiment (mg/L). Total anthocyanin content (mg cya-3-glu/L) and percent of removal (%Removal).

	L1	L1/S1 (%Removal)	L1/S2 (%Removal)	L2	L2/S1 (%Removal)	L2/S2 (%Removal)
Cyanidin-3-O-sophoriside	28.4	6.72 (76)	8.95 (68)	8.58	2.33 (73)	2.04 (76)
Cyanidin-3-O-arabinoside	0.05	n.d. * (100)	n.d. (100)	0.06	n.d. (100)	n.d. (100)
Cyanidin-3-O-glucoside	19.6	0.704 (96)	0.458 (98)	15.1	3.9 (74)	3.4 (77)
Cyanidin-3-O-rutinoside	0.894	0.246 (72)	0.313 (65)	0.503	0.21 (58)	0.196 (61)
Cyanidin-3-O-sambubioside	1.64	0.432 (74)	0.544 (67)	0.866	0.314 (64)	0.294 (66)
Malvidin-3-O-glucoside	0.078	0.045 (42)	0.046 (41)	0.066	0.047 (29)	0.048 (27)
Pelargonidin-3-O-glucoside	1.6	0.157 (90)	0.142 (91)	1.1	0.358 (67)	0.359 (67)
Pelargonidin-3-O-rutinoside	16.0	4.4 (72)	5.79 (64)	7.65	n.d. (100)	n.d. (100)
Peonidin-3-O-glucoside	0.162	0.078 (52)	0.077 (53)	0.132	0.091 (31)	0.087 (34)
TAC	58.78	17.78 (70)	19.87 (66)	25.55	13.03 (49)	3.09 (88)

* n.d.—not detected.

**Table 3:** Vibrational bands and their assignments in average spectra (Figure 5) were collected from seed samples and the literature data.

Wave Number (cm^−1^)	Literature Data	Vibrational Mode	Chemical Moiety	References
309	300	δ (C-C-C)	Glucosidic ring	[24]
491	487	Γ (C-C)	Anthocyanidins	[25]
533–540	540	Γ (C-C)	Anthocyanidins, 3-glycosides Polygalacturonic (pectic) acid	[25,26]
704	710	γ (C–O-H)	Polygalacturonic (pectic) acid	[27]
809–815	814, 818		Ferulic acid, Sinapic acid	[21]
868	868	γ (C-H)	Anthocyanidins	[22,25]
911–915	917	ν (C-O-C)	Carbohydrates, Phenylpropanoids	[28]
953, 957	953	δ (C-C-H), δ (C-O-H)	Polygalacturonic (pectic) acid	[27]
984	980	ρ (CH_2_), ν (COH)	Glucosidic link stretch	[27]
~1032	1030	C-O	Anthocyanidins	[22]
~1125	1124	νs (C-O-C)	Glucosidic link stretch	[28,29]
1182	1172, 1190	δ (OH)	Anthocyanidins	[25]
~1264	1265	δip (=CH)	cis-Lipids	[28,30]
1330–1336	1333 1325–1346	δ (CH_2_) δ (C=C–C) + δ (C–O–H) A ring	Lipids Anthocyanidins	[23,25,31]
1400	1394	-OH	Anthocyanidins	[22]
1436–1443	1441	δ (CH_2_) v (C-C)aromatic δ (C–H)	Lipids Glucosidic signal Phenolics Anthocyanidins benzopyrilium	[23,31,32,33]
1478	1471	δ (C–H) aliphatic; δ (CH_2_) or δ (CH_3_)	Lipids, Saturated branched fatty acids	[34]
1529	1520	ν (C=C) benzopyrilium, B ring and inter-ring	Anthocyanidins, Phenolics	[23,25,33]
1612–1616	1605	ν (C=C) ν (C=C) + ν (C=O) ν (C=C) B ring	Ring stretching (probably polyphenols) anthocyanidins benzopyrilium	[23,28,30,33]
1655–1659	1658	ν (C=C)	Unsaturated fatty acids; assigned to cis isomer and lignin Protein (amide I)	[28,30]
1692–1695	1690	ν (C=O)	Carboxylic acids	[28]
1732	1750	ν (C=O)	Fatty acid ester	[24]

## Data Availability

The original contributions presented in the study are included in the article/Supplementary Material, further inquiries can be directed to the corresponding author.

## Supplementary Materials

The following supporting information can be downloaded at https://www.mdpi.com/article/10.3390/foods13152334/s1. Table S1: Parameters of calibration curves, LOD and LOQ, obtained for anthocyanin standards using LC-MS analysis.
