# Supplementary material for: Untapped Potential of Side Stream Products from the Industrial Processing of Fruits: The Biosorption of Anthocyanins on Raspberry Seeds
Source: Foods. 2024 Jul 25;13(15):2334. doi: 10.3390/foods13152334 (PMC11312078; doi:10.3390/foods13152334)
Supplement: Supplementary file 1 [file foods-13-02334-s001.zip › foods-3104806-supplementary.pdf]

Article

Untapped potential of side streams products from industrial processing of fruits - Biosorption of anthocyanins on raspberry seeds

Dragana Dabić Zagorac [1], Milica Sredojević [1], Milica Fotirić Akšić [2], Ivanka Ćirić [1], Biljana Rabrenović [2], Ilinka M. Pećinar [2], and Maja Natić [3,*]

[1] Innovative Centre Faculty of Chemistry Belgrade, University of Belgrade, Studentski Trg 12-16, 11158 Belgrade, Serbia; DDZ: ddabic@chem.bg.ac.rs; MS: pantelicm@chem.bg.ac.rs; IĆ: ivankai@chem.bg.ac.rs

[2] University of Belgrade – Faculty of Agriculture, Nemanjina 6, 11080 Belgrade, Serbia; MFA: fotiric@agrif.bg.ac.rs; BR: biljanar@agrif.bg.ac.rs; IMP: ilinka@agrif.bg.ac.rs

[3] University of Belgrade – Faculty of Chemistry, Studentski Trg 12-16, 11158 Belgrade, Serbia; MN: mnatic@gmail.com

* Correspondence: MN: mnatic@gmail.com; IĆ: ivankai@chem.bg.ac.rs

Table S1. Parameters of calibration curves, LOD and LOQ obtained for anthocyanin standards using LC-MS analysis.

Anthocyanins	Intercept	Slope	R^2	Range (ppb)	LOD (ppb)	LOQ (ppb)
Cyanidin-3-O-sophoriside	-62815.6	1132.4	0.9974	250-1000	33	100
Cyanidin-3-O-arabinoside	-35274.5	962.8	0.9898	250-1000	132.7	402.2
Cyanidin-3-O-glucoside	-151080.0	1344.0	0.9973	250-1000	67.3	203.9
Cyanidin-3-O-rutinoside	-58766.0	822.1	0.9884	250-1000	141.2	427.8
Cyanidin-3-O-sambubioside	-79848.5	1226.5	0.9987	250-1000	46.2	139.9
Malvidin-3-O-glucoside	-60730.0	1502.0	0.9972	250-1000	69.6	211.0
Pelargonidin-3-O-glucoside	-234224.0	2531.0	0.9996	250-1000	24.7	74.9
Pelargonidin-3-O-rutinoside	-1133.0	14.4	0.9993	250-1000	34.2	103.5
Peonidin-3-O-glucoside	-159806.0	2345.2	0.9974	250-1000	54.7	165.8
